# Effects of mesenchymal stem cells and heparan sulfate mimetics on urethral function and vaginal wall biomechanics in a simulated rat childbirth injury model

**DOI:** 10.1007/s00192-022-05439-4

**Published:** 2023-01-20

**Authors:** Kristine Janssen, Geertruida W. van Ruiten, Niels Eijkelkamp, Margot S. Damaser, Carl H. van der Vaart

**Affiliations:** 1 Division Woman and Baby, University Medical Center Utrecht, Heidelberglaan 100, 3584CX Utrecht, the Netherlands; 2 Advanced Platform Technology Center, Louis Stokes Cleveland VA Medical Center, Cleveland, OH USA; 3 Biomedical Engineering Department, Lerner Research Institute, Cleveland Clinic, Cleveland, OH USA; 4 Center of Translational Immunology, University Medical Center Utrecht, Utrecht, the Netherlands; 5 Glickman Urological and Kidney Institute, Cleveland Clinic, Cleveland, OH USA

**Keywords:** MSCs, RGTA®, Biomechanical properties, Animal model, SUI

## Abstract

**Introduction and hypothesis:**

New treatments are needed for pelvic floor disorders. ReGeneraTing Agent® (RGTA®) is a promising regenerative therapy. Therefore, the objective of this study was to compare regenerative abilities of mesenchymal stem cells (MSCs) and RGTA® on regeneration after simulated childbirth injury in rats.

**Methods:**

Rats underwent pudendal nerve crush and vaginal distension (PNC+VD) or sham injury. Rats that underwent PNC+VD were treated intravenously with vehicle, MSCs or RGTA® 1 h, 7 days, and 14 days after surgery. Sham rats received 1 ml vehicle at all time points. After 21 days, urethral function and pudendal nerve function were tested. Vaginal tissues were harvested for biomechanical testing and histology. Biaxial testing was performed to measure tissue stiffness.

**Results:**

PNC+VD decreased urethral and pudendal nerve function compared with sham. Vaginal wall stiffness was significantly decreased in longitudinal and transverse tissue axes after PNC+VD compared with sham. MSC or RGTA® did not restore urethral or pudendal nerve function. However, MSC treatment resolved loss in vaginal wall stiffness in both tissue axes and improved collagen content within the vaginal wall. RGTA® treatment increased vaginal wall anisotropy by increasing relative stiffness in the longitudinal direction. PNC+VD (with vehicle or MSCs) enhanced elastogenesis, which was not observed after RGTA® treatment.

**Conclusions:**

Treatment with MSCs facilitated recovery of vaginal wall biomechanical properties and connective tissue composition after PNC+VD, whereas treatment with RGTA® resulted in anisotropic biomechanical changes. This indicates that MSCs and RGTA® promote different aspects of vaginal tissue regeneration after simulated childbirth injury.

**Supplementary information:**

The online version contains supplementary material available at 10.1007/s00192-022-05439-4.

## Introduction

Vaginal childbirth can result in pelvic floor disorders (PFD), including stress urinary incontinence (SUI) and pelvic organ prolapse (POP) [1]. Insufficient regeneration after damage to connective tissue, nerves, and muscles during vaginal childbirth can lead to PFD development [2].

Current treatment options for PFD are mainly surgical, but high recurrence rates and complications due to the use of vaginal mesh indicate that there is a need for the development of novel treatment options for PFD [1, 3].

Various animal models have been used to mimic PFD in humans. Simulated childbirth injury causes SUI in rats [4] and has also been shown to negatively affect uniaxial biomechanical properties and nanoscale tissue stiffness of the vaginal wall [5, 6].

Prior studies have shown that administration of mesenchymal stem cells (MSCs) prevents development of SUI in animal models of PFD. In these studies, MSC administration enhanced connective tissue repair and vascular density [7, 8]. Because of potential safety issues with stem cell-based regenerative treatments [9], cell-free regenerative treatments have been developed. One of these strategies is to promote tissue regeneration by substituting heparan sulfates (HS) in damaged tissues. ReGeneraTing Agents® (RGTA®) have been engineered to produce a nano-polysaccharide that mimics HS [10]. Systemic RGTA® treatment demonstrated enhanced tissue repair for vascular and diabetic ulcers, showing decreased time of wound healing, increased collagen content, and improved biomechanical strength of the ulcer [11]. Likewise, local injection of RGTA® improved the lesion size of acute superficial digital flexor tendonitis in racing horses and tendon repair in animal studies. The RGTA®-treated horses won significantly more races than the placebo-treated horses [12]. In addition, RGTA® enhanced healing of corneal ulcers in humans [13]. RGTA® is considered a safe product, as there is no evidence of toxicity and it is well tolerated clinically [10].

We hypothesized that simulated childbirth injury in rats, consisting of pudendal nerve crush and vaginal distention (PNC+VD), alters vaginal biomechanical properties and connective tissue composition. Administration of MSCs or RGTA® is hypothesized to promote tissue regeneration and restore urethral and pudendal nerve function, vaginal biomechanical properties, and vaginal wall connective tissue composition. Therefore, we

1. Determined the effect of simulated childbirth injury on vaginal biomechanical properties and connective tissue composition
2. Evaluated the effects of a cellular and noncellular treatment on urethral and pudendal nerve function, and on the biomechanical properties and connective tissue composition of the vaginal wall

## Materials and methods

### Treatments

Bone marrow-derived MSCs of Sprague–Dawley rats were purchased (Life Technologies, Gibco® Sprague-Dawley MSCs, cryopreserved) and cultured according to the manufacturer's instructions, as was done before in our laboratory [14]. We administered 2×10^6^ MSCs (passage 4-8) in 1 ml of phosphate buffered saline (PBS) intravenously (IV), a dose based on prior research [11]. For treatment with RGTA® OTR4131 (OTR3 Inc., Paris, France) 0.25 mg was diluted in 1 ml PBS and administered as a dose of 1 mg/kg body weight IV. This dose was based on results from previous studies [15].

### Animals

Experiments were performed in accordance with Dutch regulations and approved by the Experimental Animal Committee of the University Medical Center Utrecht (license number 2014.III.08.077). Age-matched virgin female Sprague–Dawley rats (Charles River, Maastricht, the Netherlands), aged 6–10 weeks (240–270 g), were housed in random groups with access to food and water ad libitum.

Rats were randomly allocated to four different experimental groups. Rats were anesthetized by mechanical ventilation with 2% isoflurane in air/oxygen (2:1) and received buprenorphine analgesia, 0.5 mg/kg subcutaneously.

Sham and PNC+VD surgery was performed as described previously [4, 7]. In short, the pudendal nerve was approached dorsally in the ischiorectal fossa and crushed bilaterally by compressing it twice for 30 s. VD was performed by vaginal accommodation of bougie à boule urethral dilators (24–32 Fr) followed by inflation of a modified 10-Fr Foley balloon catheter with 3 mL water, which was kept intravaginally for 4 h. Sham injury was performed by making a dorsal skin incision and using the urethral dilators with insertion and fixation of the catheter without balloon inflation. Before waking up and 1 and 2 days after surgery, rats received subcutaneous Carprofen analgesia in a subcutaneous dose of 5 mg/kg.

Rats that underwent sham surgery were treated with 1 ml vehicle (saline, 0.9% NaCl) via lateral tail vein injection 1 h, 7 days, and 14 days after surgery. At these time points, rats that underwent PNC+VD were treated with vehicle, MSCs, or RGTA®. All intravenous injections took place under 2% isoflurane ventilation anesthesia.

Twenty-one days after (sham) injury, rats were anesthetized with 2% isoflurane in air/oxygen (2:1) and received an intraperitoneal injection of urethane (1.2 g/kg). The isoflurane anesthesia was discontinued, as it impacts urethral and nerve function [7]. Urethral and pudendal nerve functional testing was performed as described previously [4]. Functional testing was performed within a time span of 30–45 min for each animal, under continuous monitoring of respiratory rate and muscle tension to assess quality of anesthesia. Following functional testing, rats were euthanized with an overdose of intracardiac Euthasol® (sodium pentobarbital, 200 mg/ml) injection and the urethra and vagina were harvested. Tissues were wrapped in saline-soaked gauze and stored at −20°C. Additional rats were anesthetized and euthanized, without undergoing urethral function testing in order to maintain anatomical integrity for histological analyses.

### Urethral function testing

All functional tests were performed in a blinded manner as described previously [7]. A suprapubic catheter (PE50) was inserted into the bladder and connected to a syringe pump and a pressure transducer. The bladder was filled with saline at a rate of 5 ml/h. After exposure of the urethra, bipolar parallel electrodes were placed to record EUS electromyography (EMG) and connected to an amplifier with bandpass frequencies of 3 Hz to 3 kHz and an electrophysiological recording system with a 10-kHz sampling rate (PowerLab 8/35, ADInstruments, Colorado Springs, CO, USA).

Leak point pressure with simultaneous recordings of EUS EMG were used to assess urethral function [7]. At approximately half bladder capacity, bladder pressure was slowly increased manually. This external pressure was removed as soon as leakage was observed at the urethral meatus. This was done 3–6 times in each animal. Thereafter, the left sensory branch of the pudendal nerve was exposed and hooked onto a bent bipolar electrode (31-gauge blunt needles, 0.8 mm apart). The skin of the genital area was brushed with a gauze and electroneurograms (ENG) were recorded approximately 5–10 times at rest and during brushing [7].

### Biomechanical testing

All biomechanical tests were performed in a blinded manner. Tissues were thawed at room temperature. The vagina was cut open along the urethra (Appendix 1c), and a square piece of tissue was obtained from the inner part, by using the cutting block (Appendix 1b) in two perpendicular directions. Prior to testing, the tissue thickness was measured using a 90° tilted microscope (Keyence® VHX-500F; Keyence Corp, Osaka, Japan; magnification ×50). Tissues were fixated with BioRakes® (5 tines, tine diameter 300 μm, tine space 1.0 mm, puncture length 2.0 mm, Appendix 1a, d, e), for which the system was calibrated. Samples were submerged in the PBS-filled water bath at 37°C. All samples were prepared, fixed, and tested in the same orientation.

Simultaneous tests using the same tension in longitudinal (x-axis) and transverse (y-axis) directions were performed using the BioTester® system and LabJoy® software after a preload of 0.1 N until the displacement was normalized. Cyclic loading was performed to levels of 4.6%, 10%, 15.7%, and 21.6% strain, with 10 cycles per strain level at a strain rate of 100% strain/min [16]. Stress was measured with 5N load cells. Data were sampled at 30 Hz. For each strain level, the last 3 out of 10 cycles were extracted and averaged.

### Vaginal wall anatomy

Vaginal wall and urethral complexes were fixed (formaldehyde 40%), embedded in OCT, and frozen and stored at −80°C. Cryosections (10 μm) were cut along the transverse direction and near sections were strained with Elastin von Gieson stain for elastin and Masson’s Trichome stain for collagen and muscle/cytoplasm.

### Data analysis

Leak point pressure as well as EUS EMG and ENG firing rate and amplitude were calculated as was done previously [4]. LPP is the difference between baseline bladder pressure and peak pressure at which urine leaks out of the urethral meatus. One-second segments of EUS EMG at baseline and peak pressure were used to determine firing rate and amplitude (MATLAB®, MathWorks Version 8.5, R2015a). Likewise, 1-s ENG segments during rest and brushing of the genital skin were selected and used to calculate firing rate and amplitude. Outcomes were calculated as the difference in firing rate and amplitude between baseline and stimulated segments: peak pressure of LPP testing for LPP and EUS EMG and clitoral brushing for ENG. Mean values per animal were utilized to create group means for statistical comparisons.

For analysis of biomechanical data, the first-order polynomial *polyfit* MATLAB® function was used to determine the slope along the loading and unloading data, and subsequently averaged to obtain a mean slope representing tissue stiffness. This was done for both the longitudinal and the transverse axes by application of a custom-made MATLAB script (MathWorks Version 8.5, R2015a). For each strain level, the last 3 out of 10 cycles were extracted and averaged. Tangent modulus was determined by calculating the slope over the last 1% strain interval of the curve, which is the slope over the linear region of the curve. To assess anisotropy of vaginal biomechanics, we defined the Anisotropy Index (AI) as the mean longitudinal tangent modulus divided by mean transverse tangent modulus for each specimen tested.

Tangent moduli were used as our primary outcome for vaginal biomechanical properties. Based on previous studies in other species [17, 18], a mean difference of vaginal wall stiffness of 30% between injured and sham injured animals was used to calculate sample size. In order to demonstrate this difference at a significance level of 0.05 and power of 0.9, a minimum of seven animals were required in each group.

Statistical analysis was performed using Prism® version 8.1 (GraphPad Software Inc., La Jolla, CA, USA) and results are shown as mean ± standard error of the mean (SEM). Urethral function data were analyzed using a Kruskal–Wallis test with a Dunn’s multiple comparison post hoc test. Two-way repeated measures ANOVA with Bonferroni correction were used to evaluate differences in tangent moduli. Mixed-model analysis with Bonferroni correction was used to assess vaginal wall anisotropy between treatment groups. *p*<0.05 was considered to indicate a statistically significant difference between groups in all cases.

Histology was assessed qualitatively by a blinded investigator. The density, length, thickness, location, organization, and orientation of the elastin fibers within the vaginal wall were evaluated on the Elastin van Gieson slides. Masson’s Trichome slides were evaluated for collagen content and collagen fiber direction within the vaginal wall.

## Results

Nineteen rats underwent sham injury. Mortality occurred in 3 rats in this group due to the initial anesthesia. Functional outcome data were available for all rats except for one rat in the MSC group, because of mortality after urethane injection prior to LPP testing. Thirty-two rats underwent PNC+VD, of which 15 rats received vehicle treatment, 9 rats MSC treatment, and 8 rats RGTA® treatment. An additional 12 rats (*n*=3 per experimental group) underwent (sham) injury and received either vehicle, MSC, or RGTA® treatment and were used for histological outcome testing.

### Urethral function

**Fig. 1:**
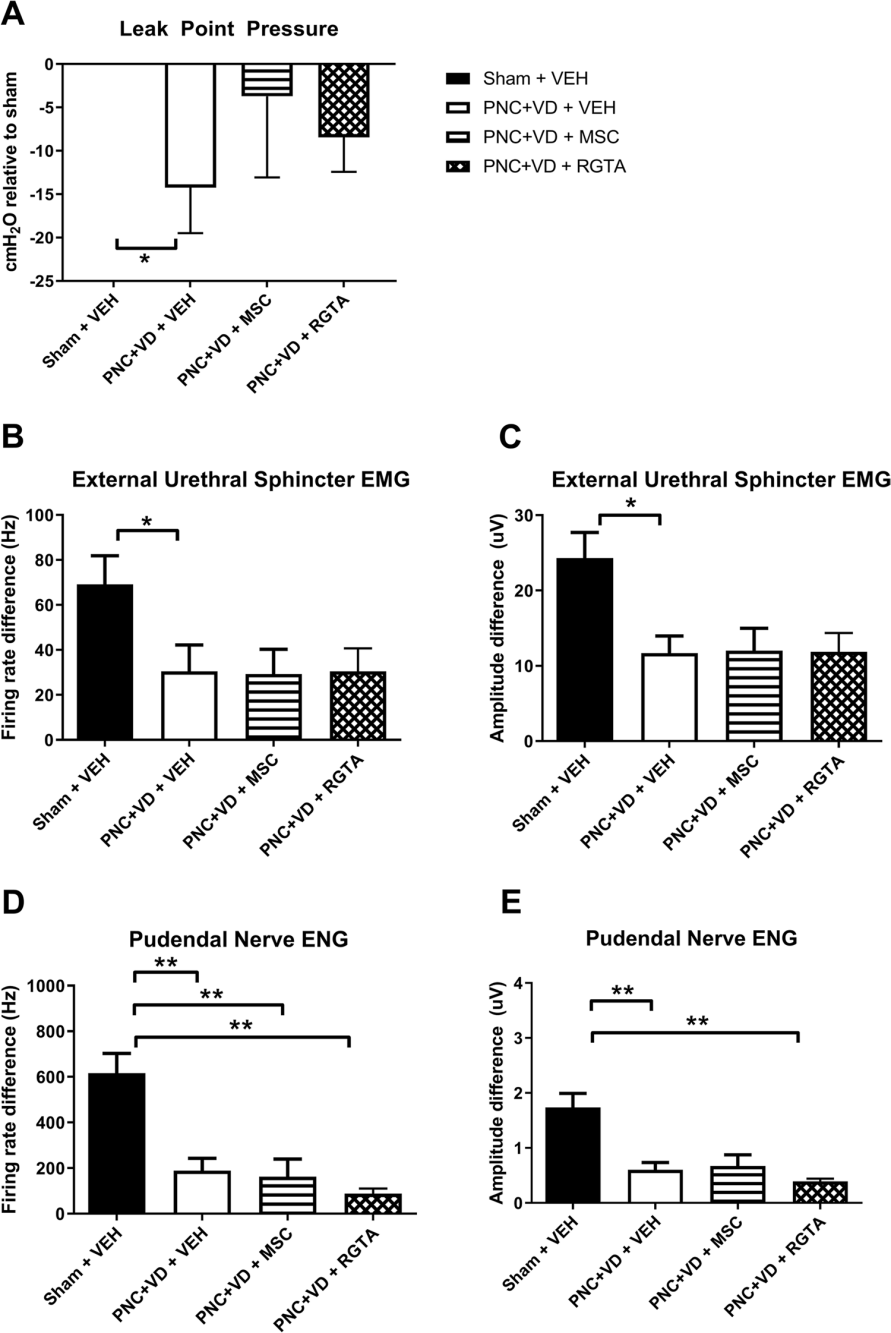
Functional outcomes. Bar charts represent urethral and pudendal nerve function outcome data after (sham) pudendal nerve crush and vaginal distension (*PNC+VD*) and treatment with vehicle (*VEH*), mesenchymal stem cells (*MSCs*), or ReGeneraTing Agent® (*RGTA*). *Black bars* indicate the sham injury and vehicle-treated group (Sham + VEH, *n*=16), *white bars* indicate the pudendal nerve crush and vaginal distension (PNC+VD) injury, and the vehicle-treated group (PNC+VD + VEH, *n*=15), *striped bars* indicate the MSC-treated group (PNC+VD + MSC, *n*=9), and *checked bars* indicate the RGTA-treated group (PNC+VD + RGTA *n*=8). **A** Leak point pressure data are displayed in cm H_2_O as mean difference (SEM) compared with the mean of the sham injury group. External urethral sphincter electromyography (*EMG*) data are displayed as difference in **B** firing rate (Hz, ) and **C** amplitude (μV) between baseline and leak point pressure testing. Pudendal nerve electroneurography (*ENG*) data are displayed as difference in **D** firing rate (Hz) and **E** amplitude (μV) between baseline and brushing of the clitoral skin. Each bar represents the mean ± standard error of the mean of data from 8 to 19 animals. *p<0.05; **p<0.01

Leak point pressure was significantly reduced 21 days after PNC+VD compared with sham injury (Fig. 1A; *p*=0.013). Injection of either 2x10^6^ MSCs or 1 mg/kg RGTA® 1 h, 7, and 14 days after PNC+VD did not significantly change LPP 21 days after surgery. Urethral function as measured with EUS EMG firing rate and amplitude decreased significantly after PNC+VD compared with sham injury (Fig. 1B, *p*=0.033, and Fig. 1C, *p*=0.011). MSC or RGTA® treatment did not significantly improve EUS EMG. Pudendal nerve function as measured with ENG of the pudendal nerve during brushing of the genital skin was decreased after PNC+VD compared with sham (Fig. 1D, *p*=0.0030, and Fig. 1E; *p*=0.0042). MSC or RGTA® treatment did not prevent this loss of function. In this study, with the doses tested, MSC or RGTA® were not able to restore impaired urethral function after childbirth injury.

### Biomechanical properties of the vaginal wall

Seven rats in the sham injury group and 8 in the PNC+VD and vehicle treatment group were used to optimize the biaxial testing protocol and thus data could not be used for further analyses (e.g., the use of different load cells resulted in deviating, nonrepeatable measures). Biomechanical data were available for the following numbers of animals: sham injury—*n*=12, PNC+VD and vehicle treatment—*n*=7, PNC+VD and MSC treatment—*n*=9, and PNC+VD and RGTA® treatment—*n*=7. Data from one injured rat treated with RGTA® had to be excluded owing to insufficient BioRake® fixation, which resulted in strongly deviating biomechanical behavior.

All measured samples demonstrated a nonlinear stress–strain curve, starting with a toe region followed by a small linear elastic region from which the tangent modulus was derived. Ten cycles were sufficient for the viscoelastic tissue to exhibit steady-state mechanics. Therefore, the last three cycles were used for further analysis. Appendix 2 shows a representative example of a stress–strain curve.

**Fig. 2:**
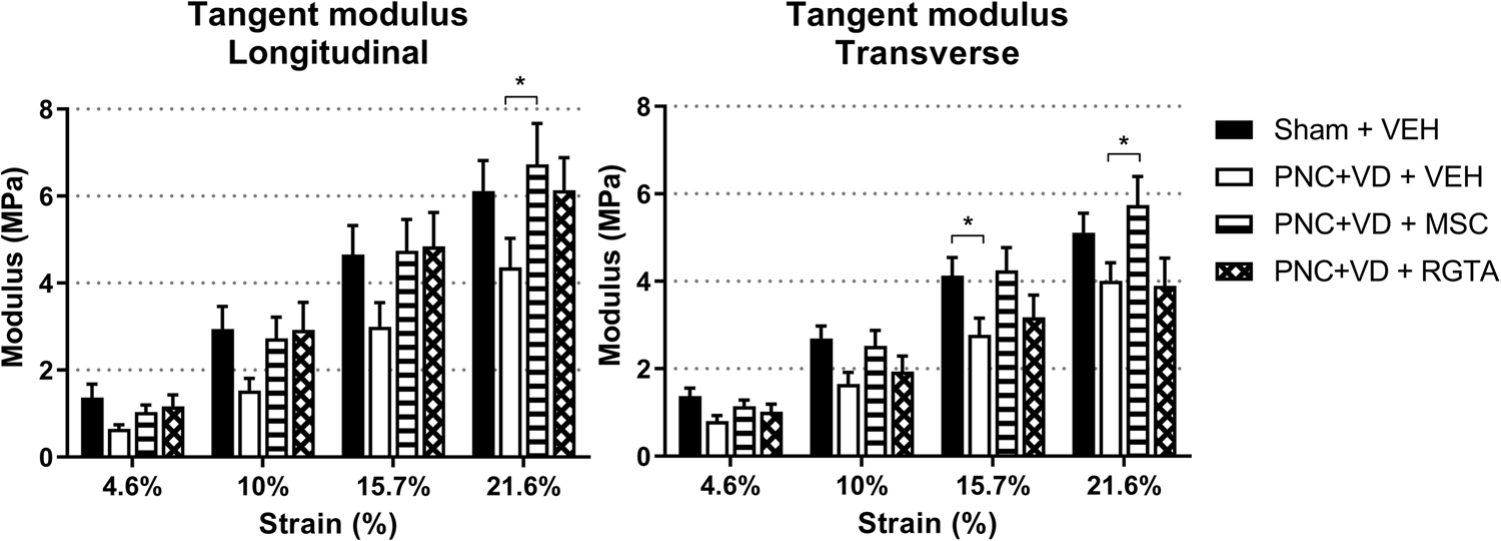
Mean tangent moduli per strain level. Bar charts represent tangent moduli in MPa as a result of biaxial biomechanical testing of the rat vaginal wall after (sham) pudendal nerve crush and vaginal distension (PNC+VD) and treatment with vehicle (VEH), mesenchymal stem cells (MSC), or ReGeneraTing Agent® (RGTA). *Black bars* indicate the sham injury and vehicle-treated group (Sham + VEH, *n*=12), *white bars* indicate the injury and vehicle-treated group (PNC+VD + VEH, *n*=7), *striped bars* indicate the MSC-treated group (PNC+VD + MSC, *n*=9), and *checked bars* indicate the RGTA-treated group (PNC+VD + RGTA, *n*=7). Each bar represents the mean ± standard error of the mean of data from 8 to 19 animals. *p<0.05

The PNC+VD animals had lower mean tangent moduli in both longitudinal and transverse directions 21 days after injury, compared with sham-injured animals (Fig. 2), although this was significantly different only at 15.7% strain in the transverse direction (Fig. 2, *p*=0.046). These findings indicate that biaxial biomechanical properties of the vaginal wall were impacted by PNC+VD, resulting in reduced stiffness of the vaginal wall.

Injection of MSCs increased mean tangent modulus compared with vehicle treatment after PNC+VD in the longitudinal (*p*=0.026) and transverse (*p*=0.020) directions at 21.6% strain (Fig. 2). RGTA® treatment did not significantly improve tissue stiffness compared with vehicle treatment after PNC+VD.

**Fig. 3:**
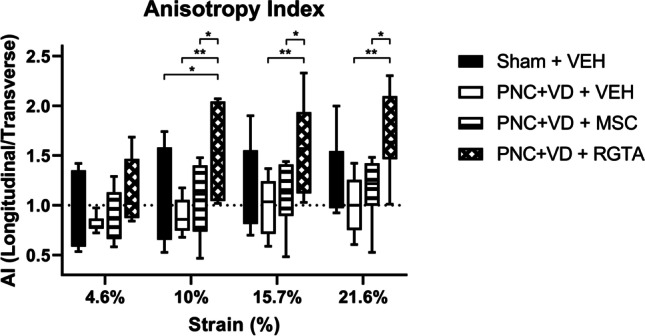
Anisotropy indices per strain level. Boxplots represent anisotropy indices (AIs), which were calculated by dividing tangent moduli in the longitudinal direction by tangent moduli in the transverse direction of the rat vaginal wall after (sham) pudendal nerve crush and vaginal distension (PNC+VD) and treatment with vehicle, mesenchymal stem cells (MSCs), or ReGeneraTing Agent® (RGTA). *Black bars* indicate the sham injury and vehicle (VEH)-treated group (Sham + VEH, *n*=12), *white bars* indicate the injury and vehicle-treated group (PNC+VD + VEH, *n*=7), *striped bars* indicate the MSC-treated group (PNC+VD MSC, *n*=9), and *checked bars* indicate the RGTA-treated group (PNC+VD + RGTA, *n*=7). Each box represents the median ± interquartile ranges of data from 8 to 19 animals. *Asterisks* indicate statistical significant differences between treatment groups within each strain level. *p<0.05, **p<0.01

As we tested biaxial biomechanical properties, we were able to explore the anisotropic behavior for all experimental groups. By calculating anisotropy indices (AIs), we compared mean tangent moduli between longitudinal and transverse tissue axes. Treatment with RGTA® increased vaginal wall anisotropy compared with vehicle treatment at 10% (*p*=0.049), 15.7% (*p*=0.003), and 21.6% (*p*=0.0014) strain levels (Fig. 3). Also, treatment with RGTA® increased vaginal wall anisotropy compared with MSC treatment at 10% (*p*=0.021) and 15.7% (*p*=0.039) strain levels (Fig. 3). At 10% strain, RGTA® treatment significantly increased vaginal wall anisotropy compared with the sham-injured vaginal wall (*p*=0.049, Fig. 3). This indicates increased vaginal wall anisotropy with increased stiffness in the longitudinal direction compared with the transverse direction after RGTA® treatment.

### Vaginal wall histology

**Fig. 4:**
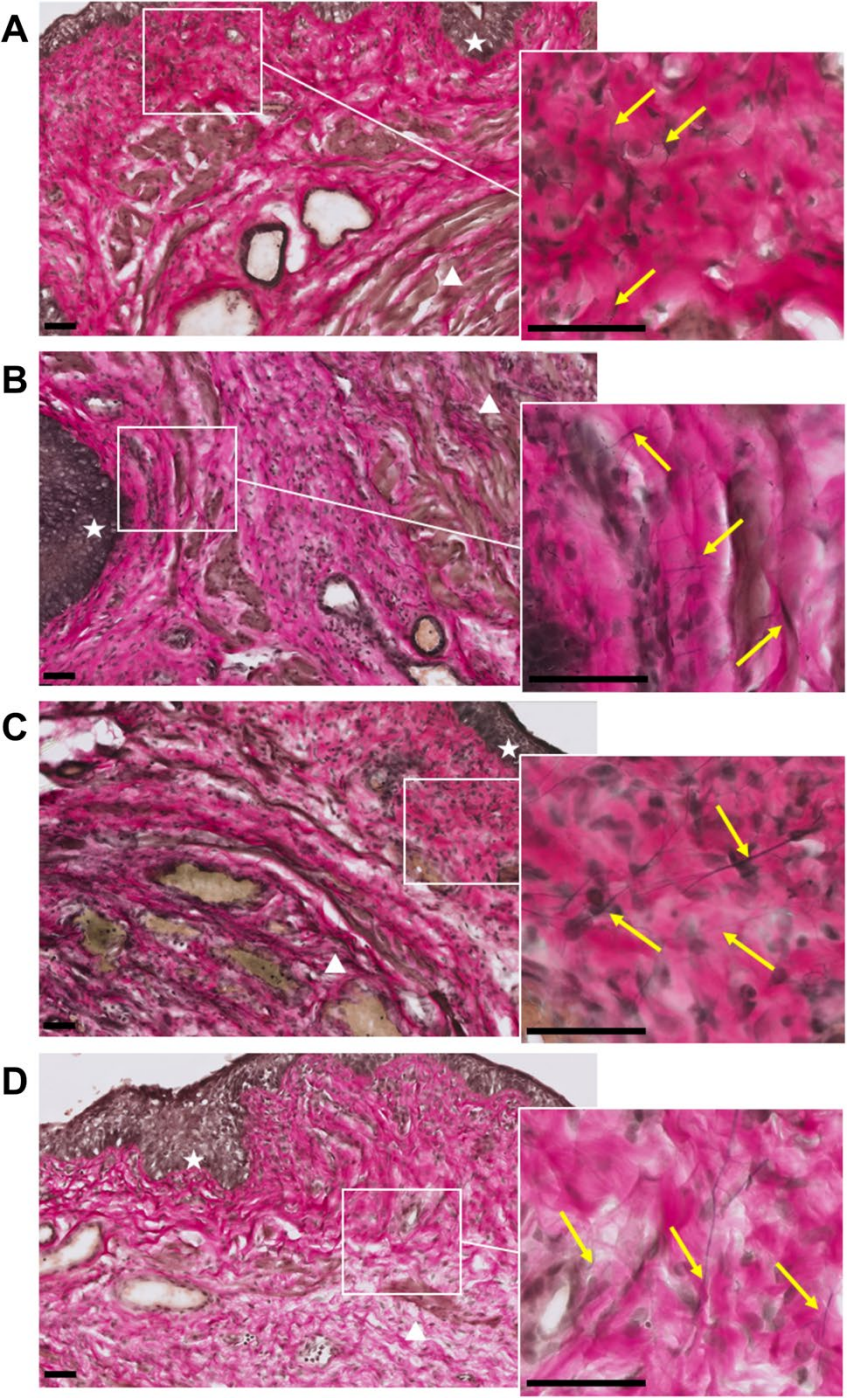
Elastin fibers within the vaginal wall. Images display representative histological examples of transverse sectioned vaginal walls (epithelium indicated by the *white star* and urethral sphincter complex by the *white triangle*), stained with Elastin von Gieson stain. **B** Pudendal nerve crush and vaginal distension (PNC+VD) resulted in increased density of elastin fibers (*yellow arrows*) compared with **A** sham injury, indicating increased vaginal wall elastogenesis after childbirth injury. Likewise, the density of elastin fibers increased after **C** mesenchymal stem cells (MSC) treatment compared with sham. **D** ReGeneraTing Agent® (RGTA®) treatment resulted in sparse elastin fibers, comparable with the sham group. *white triangles* indicate the urethral sphincter complex. *Black bars* are scale bars, indicating 50 μm

In all Elastin van Gieson-stained sections, the most prominent location of elastin fibers was found within the attachment between the urethra and vaginal wall and along the vaginal wall smooth muscle. PNC+VD resulted in increased density of elastin fibers compared with sham injury, indicating increased vaginal wall elastogenesis after childbirth injury (Fig. 4A, B). Likewise, the density of elastin fibers increased after MSC treatment compared with sham (Fig. 4C). In contrast, RGTA® treatment resulted in sparse elastin fibers, comparable with the sham group (Fig. 4D). Elastin location, thickness, length, and organization were heterogeneous and did not differ between groups.

**Fig. 5:**
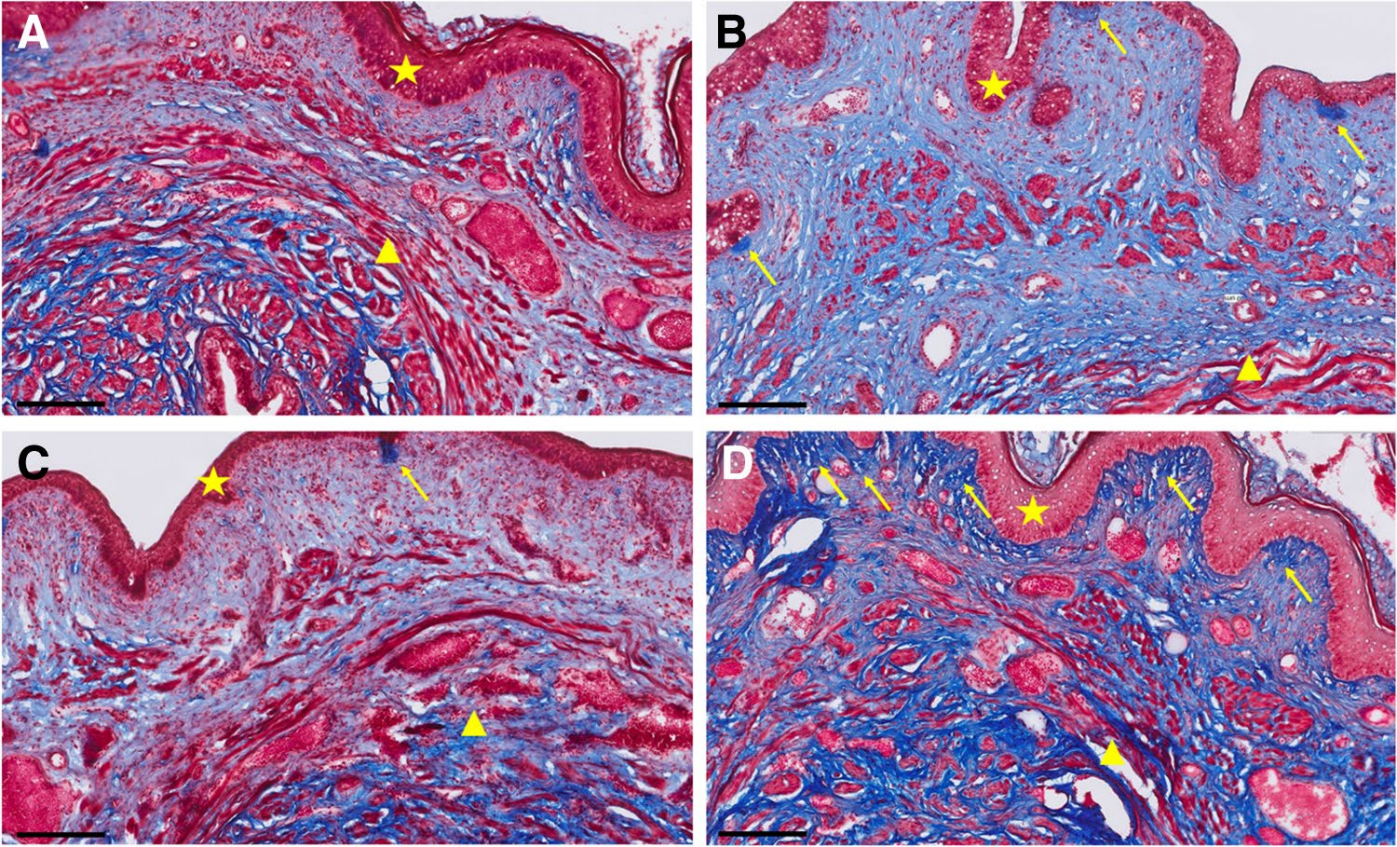
Collagen and muscle content of the vaginal wall. Images display representative examples of histological slides stained with Masson’s Trichome. *Blue* indicates collagen and *red* indicates cytoplasm and nuclei. The vaginal epithelium is indicated by a *yellow star* and the urethral sphincter complex by a *yellow triangle*. **B** Pudendal nerve crush and vaginal distension (PNC+VD) resulted in higher collagen content within the vaginal wall, compared with **A** the sham group. **C** After mesenchymal stem cells (MSC) treatment, collagen content was comparable with sham. **D** The RGTA®-treated group consistently showed increased collagen content, which was comparable with PNC+VD. Likewise, after PNC+VD (**B**), we found foci with increased density of collagen (*yellow arrows*) directly under the vaginal epithelium, which we did not observe in the sham-injured animals (**A**). These foci were less pronounced after MSC treatment (**C**), but ReGeneraTing Agent® (RGTA®) treatment (**D**) lead to a clear increase in collagen density in the subepithelium. These findings indicated collagen deposition within the vaginal wall. The collagen fiber direction was heterogenous and similar throughout the samples. *Black bars* are scale bars, indicating 200 μm

Microscopic analysis of Masson’s Trichome-stained slides revealed a remarkably higher collagen content in the PNC+VD group and the RGTA®-treated groups consistently (Fig. 5B, D), compared with sham animals and animals treated with MSCs (Fig. 5A, C). Likewise, in the samples of PNC+VD animals, we found localized increased density of collagen in the subepithelium, which we did not observe in the sham-injured animals (Fig. 5A, B). these foci were less pronounced after MSC treatment (Fig. 5C), but RGTA® treatment lead to a clear increase in collagen density in the subepithelium (Fig. 5D). the collagen fiber direction was heterogeneous and similar throughout the samples.

## Discussion

We hypothesized that simulating birth injury in rats, with PNC+VD might cause alterations in the biomechanical properties of the vaginal wall. Additionally, we evaluated the effects of two experimental regenerative therapies, one cellular and one noncellular. We found that simulated childbirth injury causes a decrease in vaginal wall stiffness. MSC treatment improves vaginal wall stiffness in both longitudinal and transverse directions. In contrast, treatment with RGTA® increased vaginal tissue anisotropy, with higher stiffness in the longitudinal direction compared with the transverse direction. Thus, MSC treatment restores the vaginal biomechanical properties after childbirth injury, whereas RGTA® treatment only increases vaginal wall anisotropy. Histological evaluation suggests that MSC treatment might restore the collagen content, resulting in biomechanical properties similar to sham-injured animals. This effect is not seen after RGTA treatment. In addition, we observed increased elastogenesis compared with sham after PNC+VD and either vehicle or MSC treatment. In the RGTA® treatment group we did not observe this increase in elastogenesis.

Our results show that simulated childbirth injury with PNC+VD causes impairment in vaginal biomechanical properties. The observed decreased stiffness of the vaginal wall after injury is in line with the results of other childbirth injury animal studies [18, 19]. However, others did not show sustained injury in rats, as vaginal biomechanical properties are comparable with virgin values 4 weeks postpartum [19]. It should be noted that these studies were performed in pregnant animals and adaptations during pregnancy may protect against damage to the pelvic floor during delivery [18]. Additionally, rat pup delivery provides less of an injury than vaginal balloon distension, which is meant to mimic the greater damage occurring in human vaginal delivery.

We used biaxial testing instead of uniaxial testing to mimic the complex tissue behavior in vivo, which better represents the biomechanical characteristics of soft tissues [20]. Biaxial testing protocols have previously been used to study anisotropic behavior of the vaginal wall [21]. Recently Huntington et al. have shown, using biaxial biomechanical testing, that the rat vaginal wall was significantly anisotropic [21]. Similar to our study, this only became apparent at higher strain levels, above 20% strain. In contrast to our study, the vaginal wall was stiffer in the circumferential diameter than the longitudinal diameter [21]. An explanation for different findings is the use of different biaxial testing methods, different strains, and different methods of tissue preparation.

To assess the collagen content within the vaginal wall, we used Masson’s Trichome stain, which is a common method estimating collagen content in tissues [22], and found increased collagen content after PNC+VD, compared with sham. Similar results were observed in vivo by Knight et al., they showed that parous ewes had a greater amount of collagen content within the vaginal wall than nulliparous animals [17]. Moreover, we found localized foci of increased collagen density after PNC+VD. Disrupted collagen remodeling after injury, when collagen deposition exceeds degradation, indicates fibrosis [23]. This is likely the result of VD injury to the vaginal wall, as local parenchymal cell destruction leads to an inflammatory response and remodeling of the connective tissue, which initiates local fibrosis [23].

Our data show a statistically significant negative impact of PNC+VD on urethral and pudendal nerve function, as measured with LPP, EUS EMG, and pudendal nerve ENG. However, we did not observe a statistically significant improvement of any of these measurements after treatment with MSCs. In contrast, previous studies have shown a significant improvement of urethral function after treatment with either one or multiple doses of MSCs [7, 8, 24]. Factors that may contribute to these findings are use of different MSCs, multiple (four groups) group comparison, and increased variability in outcomes observed in this study. However, when assessing absolute data, effect size of MSC treatment on LPP is similar to that of a previous study [24].

We found that MSCs improved the biomechanical properties of the vaginal wall. Our histological analyses suggest that this might be due to improved connective tissue composition. Collagen and elastin are important extracellular matrix (ECM) components contributing to the biomechanical behavior of soft tissues [25]. Collagen within pelvic floor tissues provides the tissues with tensile strength, whereas elastin impacts tissue compliance [25]. After injury, a cascade of structural changes in the ECM occur [10]. Fibroblasts are crucial for maintaining the tissues’ mechanical properties, as these are the cells responsible for repair and remodeling of the ECM after tissue injury. They secrete ECM proteins and remodel their environment adapting to chemical and mechanical influences [26]. MSCs can differentiate into healthy fibroblasts that adhere and synthesize ECM components in order to repair damaged ECM after injury [27]. Moreover, MSCs have the ability to secrete growth and immunomodulatory factors that enhance tissue healing by stimulation of cell migration, proliferation, neovascularization, and regulate collagen and elastin deposition [28]. Finally, MSCs can increase elastic fiber density in the urethra after PNC+VD [7, 24]. These capacities of MSCs may explain our findings of improved collagen composition of the vaginal wall after MSC treatment, compared with vehicle treatment.

Because our previous studies showed that systemically administered MSCs promote tissue regeneration after simulated birth injury [24], we used systemic administration of MSCs in this study. Others have shown that arterial systemic injection of stem cells is more effective after birth injury than local injection [29]. Further research is needed to optimize routes of administration, also taking into account side effects or complications of the systemic injection of MSCs.

With regard to the clinical application of (bone marrow-derived) MSCs, there are still important limitations due to safety issues and determining optimal timing, treatment, and sources of stem cells [9]. Therefore, we tested RGTA®, which is readily available and safe in humans [11, 13]. Unfortunately, this regenerating treatment was not effective for vaginal regeneration. We found that RGTA® only increased vaginal wall anisotropy, indicating that the vagina becomes stiffer in the longitudinal direction than in the transverse direction. Besides, we found increased collagen content and density in the subepithelium and decreased elastin density after RGTA® treatment. Others showed that RGTA® increases collagen I and III production in the short and long term after skin burns [30]. Tong et al. studied the effects of the systemic treatment of RGTA® diabetic skin ulcers and found decreased ulceration, an increase in collagen I production, and increased biomechanical ulcer strength [11]. It can be postulated that RGTA® improves biomechanical properties only in one direction because it enhances collagen I fiber production. Also, the observed increased collagen fiber density within the subepithelium may play a role in these alterations. We did not show significant differences in collagen fiber direction; however, others have shown that healthy vaginal wall demonstrates greater collagen alignment in the longitudinal direction compared with the injured vaginal wall, which is associated with increased vaginal wall stiffness in the direction of the applied force.

Although we found increased anisotropy after RGTA® treatment, we did not find a statistically significant treatment effect for either urethral or pudendal nerve function or vaginal wall stiffness in the transverse direction. An explanation for the partial effect on biomechanical properties after RGTA® could be explained by inadequate HS dose optimization [13]. The number of HS binding sites in ECM is limited and excess RGTA® can remove growth factors from the ECM, which may cause limited availability of growth factors needed for tissue regeneration [13].

A limitation of this study is the lack of molecular outcomes to help explain the biomechanical results. With the histological analysis of the vaginal wall microstructure, we provided a starting point to explain the changes in biomechanical findings, but the underlying mechanisms (collagen/elastin crosslinking, types of collagen, fiber direction) are more complex and need to be further investigated [17, 20]. Furthermore, we used an animal model for vaginal wall damage and tested the effects of regenerative treatments on vaginal wall function, measured with vaginal biomechanical properties. It is unknown how this correlates with the development of pelvic floor dysfunction in the long term. Another limitation is that we were not able to repeat prior results showing regeneration with MSCs of urethral function in this model, perhaps because we used the purchased rat MSCs in this study, in contrast to prior studies, which used rat bone marrow-derived MSCs harvested in the laboratory [7, 24]. We did not perform additional characterization of the purchased batches of MSCs, as these cells had been used by colleagues in the same laboratory before, showing adequate MSC characterization and promising regenerative capacities [14]. Finally, we did not research whether RGTA® or MSCs reached the vagina or if their mechanisms of action are based on systemic paracrine effects.

In summary, simulated childbirth injury decreases vaginal wall stiffness and our results suggest that treatment with MSCs might facilitate biomechanical and connective tissue recovery of the vaginal wall 3 weeks after simulated birth injury, whereas treatment with RGTA® only resulted in anisotropic changes.

## Supplementary Information

Fig. 6 BioTester® system. **A**. BioRakes®. **B** Cutting block with two blades positioned at a distance of 10 mm apart. **C** Schematic representation of a vagina with urethra: *1* the vagina is cut along its length at the urethra, flipped 90° counter clockwise, and unfolded so that the epithelium is on top; *2* by using the cutting block in two perpendicular directions, a square specimen of 10×10 mm is obtained; *3* position of BioRakes® fixation. **D** Vaginal specimen mounted in BioRakes®. **E** Specimen positioned in water bath before testing. The x-axis represents the longitudinal tissue axis (*magenta*); the y-axis represents the transverse tissue axis (*cyan*)

High resolution image (TIF 1900 kb)

Fig. 7 Representative example of a stress–strain curve. All samples demonstrated nonlinear curves in both longitudinal (*magenta*, x-axis) and transverse (*cyan*, y-axis) tissue axes. *Red arrows* indicate the starting point of the last three loading cycles, which exhibit a steady state and were used for further analysis

High resolution image (TIF 2006 kb)

## References

[1] Vergeldt TF, Weemhoff M, IntHout J, Kluivers KB (2015). Risk factors for pelvic organ prolapse and its recurrence: a systematic review. Int Urogynecol J.

[2] Bortolini MA, Drutz HP, Lovatsis D, Alarab M (2010). Vaginal delivery and pelvic floor dysfunction: current evidence and implications for future research. Int Urogynecol J.

[3] Syed KK, Consolo MJ, Gousse AE (2021). Anterior vaginal wall prolapse repair and the rise and fall of transvaginal mesh. Did we come full circle? A historical perspective. Urology.

[4] Jiang HH, Pan HQ, Gustilo-Ashby MA, Gill B, Glaab J, Zaszczurynski P (2009). Dual simulated childbirth injuries result in slowed recovery of pudendal nerve and urethral function. Neurourol Urodyn.

[5] Alperin M, Feola A, Meyn L, Duerr R, Abramowitch S, Moalli P (2010). Collagen scaffold: a treatment for simulated maternal birth injury in the rat model. Am J Obstet Gynecol.

[6] Paul K, Darzi S, Del Borgo MP, Cousins FL, Werkmeister JA, Gargett CE (2021). Vaginal delivery of tissue engineered endometrial mesenchymal stem/stromal cells in an aloe vera-alginate hydrogel alleviates maternal simulated birth injury. Appl Mater Today.

[7] Deng K, Lin DL, Hanzlicek B, Balog B, Penn MS, Kiedrowski MJ (2015). Mesenchymal stem cells and their secretome partially restore nerve and urethral function in a dual muscle and nerve injury stress urinary incontinence model. Am J Physiol Renal Physiol.

[8] Sadeghi Z, Isariyawongse J, Kavran M, Izgi K, Marini G, Molter J (2016). Mesenchymal stem cell therapy in a rat model of birth-trauma injury: functional improvements and biodistribution. Int Urogynecol J.

[9] Lalu MM, McIntyre L, Pugliese C, Fergusson D, Winston BW, Marshall JC (2012). Safety of cell therapy with mesenchymal stromal cells (SafeCell): a systematic review and meta-analysis of clinical trials. PLoS One.

[10] Barritault D, Gilbert-Sirieix M, Rice KL, Sineriz F, Papy-Garcia D, Baudouin C (2017). RGTA® or ReGeneraTing agents mimic heparan sulfate in regenerative medicine: from concept to curing patients. Glycoconj J.

[11] Tong M, Tuk B, Shang P, Hekking IM, Fijneman EM, Guijt M (2012). Diabetes-impaired wound healing is improved by matrix therapy with heparan sulfate glycosaminoglycan mimetic OTR4120 in rats. Diabetes.

[12] Jacquet-Guibon S, Dupays AG, Coudry V, Crevier-Denoix N, Leroy S, Sineriz F (2018). Randomized controlled trial demonstrates the benefit of RGTA® based matrix therapy to treat tendinopathies in racing horses. PLoS One.

[13] Aifa A, Gueudry J, Portmann A, Delcampe A, Muraine M (2012). Topical treatment with a new matrix therapy agent (RGTA) for the treatment of corneal neurotrophic ulcers. Invest Ophthalmol Vis Sci.

[14] Van Velthoven CT, Sheldon RA, Kavelaars A, Derugin N, Vexler ZS, Willemen HL (2013). Mesenchymal stem cell transplantation attenuates brain injury after neonatal stroke. Stroke.

[15] Zuijdendorp HM, Smit X, Blok JH, Caruelle JP, Barritault D, Hovius SE (2008). Significant reduction in neural adhesions after administration of the regenerating agent OTR4120, a synthetic glycosaminoglycan mimetic, after peripheral nerve injury in rats. J Neurosurg.

[16] Sommer G, Schriefl AJ, Andra M, Sacherer M, Viertler C, Wolinski H (2015). Biomechanical properties and microstructure of human ventricular myocardium. Acta Biomater.

[17] Knight KM, Moalli PA, Nolfi A, Palcsey S, Barone WR, Abramowitch SD (2016). Impact of parity on ewe vaginal mechanical properties relative to the nonhuman primate and rodent. Int Urogynecol J.

[18] Alperin M, Feola A, Duerr R, Moalli P, Abramowitch S (2010). Pregnancy- and delivery-induced biomechanical changes in rat vagina persist postpartum. Int Urogynecol J.

[19] Feola A, Moalli P, Alperin M, Duerr R, Gandley RE, Abramowitch S (2011). Impact of pregnancy and vaginal delivery on the passive and active mechanics of the rat vagina. Ann Biomed Eng.

[20] Abramowitch SD, Feola A, Jallah Z, Moalli PA (2009). Tissue mechanics, animal models, and pelvic organ prolapse: a review. Eur J Obstet Gynecol Reprod Biol.

[21] Huntington A, Rizzuto E, Abramowitch S, Del Prete Z, De Vita R (2019). Anisotropy of the passive and active rat vagina under biaxial loading. Ann Biomed Eng.

[22] Zhang J, Zhu Y, Pan L, Xia H, Ma J, Zhang A (2019). Soy isoflavone improved female sexual dysfunction of mice via endothelial nitric oxide synthase pathway. Sex Med.

[23] Weiskirchen R, Weiskirchen S, Tacke F (2019). Organ and tissue fibrosis: molecular signals, cellular mechanisms and translational implications. Mol Asp Med.

[24] Janssen K, Lin DL, Hanzlicek B, Deng K, Balog BM, van der Vaart CH (2019). Multiple doses of stem cells maintain urethral function in a model of neuromuscular injury resulting in stress urinary incontinence. Am J Physiol Renal Physiol.

[25] Rynkevic R, Martins P, Andre A, Parente M, Mascarenhas T, Almeida H (2019). The effect of consecutive pregnancies on the ovine pelvic soft tissues: link between biomechanical and histological components. Ann Anat.

[26] Ruiz-Zapata AM, Kerkhof MH, Zandieh-Doulabi B, Brolmann HA, Smit TH, Helder MN (2014). Functional characteristics of vaginal fibroblastic cells from premenopausal women with pelvic organ prolapse. Mol Hum Reprod.

[27] Lee CH, Shah B, Moioli EK, Mao JJ (2010). CTGF directs fibroblast differentiation from human mesenchymal stem/stromal cells and defines connective tissue healing in a rodent injury model. J Clin Invest.

[28] Jeon YK, Jang YH, Yoo DR, Kim SN, Lee SK, Nam MJ (2010). Mesenchymal stem cells' interaction with skin: wound-healing effect on fibroblast cells and skin tissue. Wound Repair Regen.

[29] Mori da Cunha M, Giacomazzi G, Callewaert G, Hympanova L, Russo F, Vande Velde G (2018). Fate of mesoangioblasts in a vaginal birth injury model: influence of the route of administration. Sci Rep.

[30] Garcia-Filipe S, Barbier-Chassefiere V, Alexakis C, Huet E, Ledoux D, Kerros ME (2007). RGTA OTR4120, a heparan sulfate mimetic, is a possible long-term active agent to heal burned skin. J Biomed Mater Res A.

