# Supplementary figures and images for: Effects of mesenchymal stem cells and heparan sulfate mimetics on urethral function and vaginal wall biomechanics in a simulated rat childbirth injury model
Source: Int Urogynecol J. 2023 Jan 20;34(7):1635–44. doi: 10.1007/s00192-022-05439-4 (PMC10287815; doi:10.1007/s00192-022-05439-4)

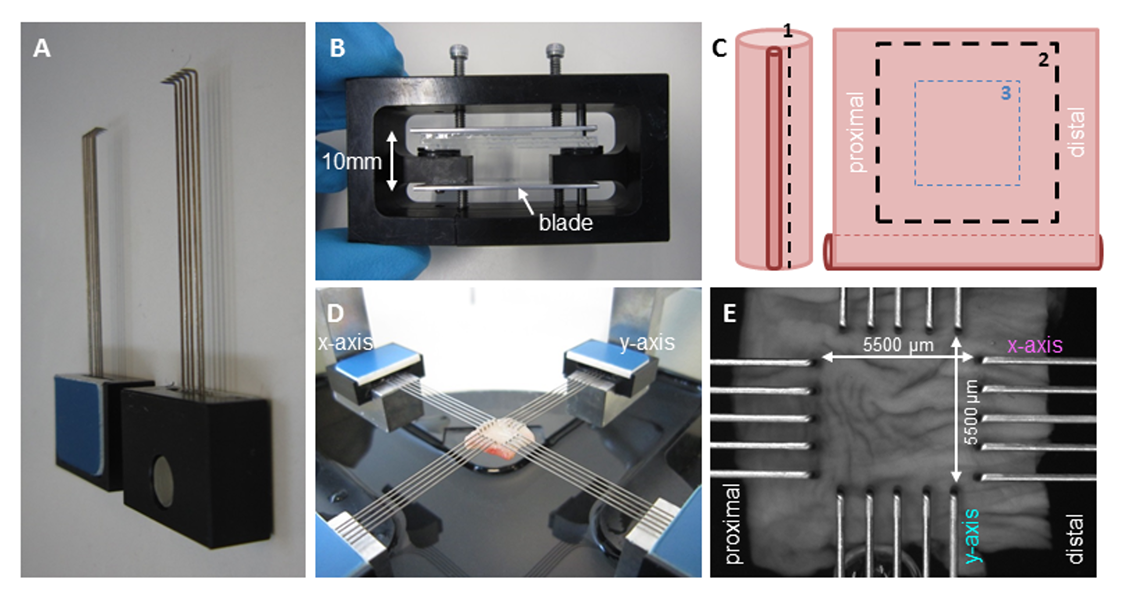

Supplement: Supplementary file 1 — BioTester® system. A. BioRakes®. B Cutting block with two blades positioned at a distance of 10 mm apart. C Schematic representation of a vagina with urethra: 1 the vagina is cut along its length at the urethra, flipped 90° counter clockwise, and unfolded so that the epithelium is on top; 2 by using the cutting block in two perpendicular directions, a square specimen of 10×10 mm is obtained; 3 position of BioRakes® fixation. D Vaginal specimen mounted in BioRakes®. E Specimen positioned in water bath before testing. The x-axis represents the longitudinal tissue axis (magenta); the y-axis represents the transverse tissue axis (cyan) [file 192_2022_5439_Fig6_ESM.png]

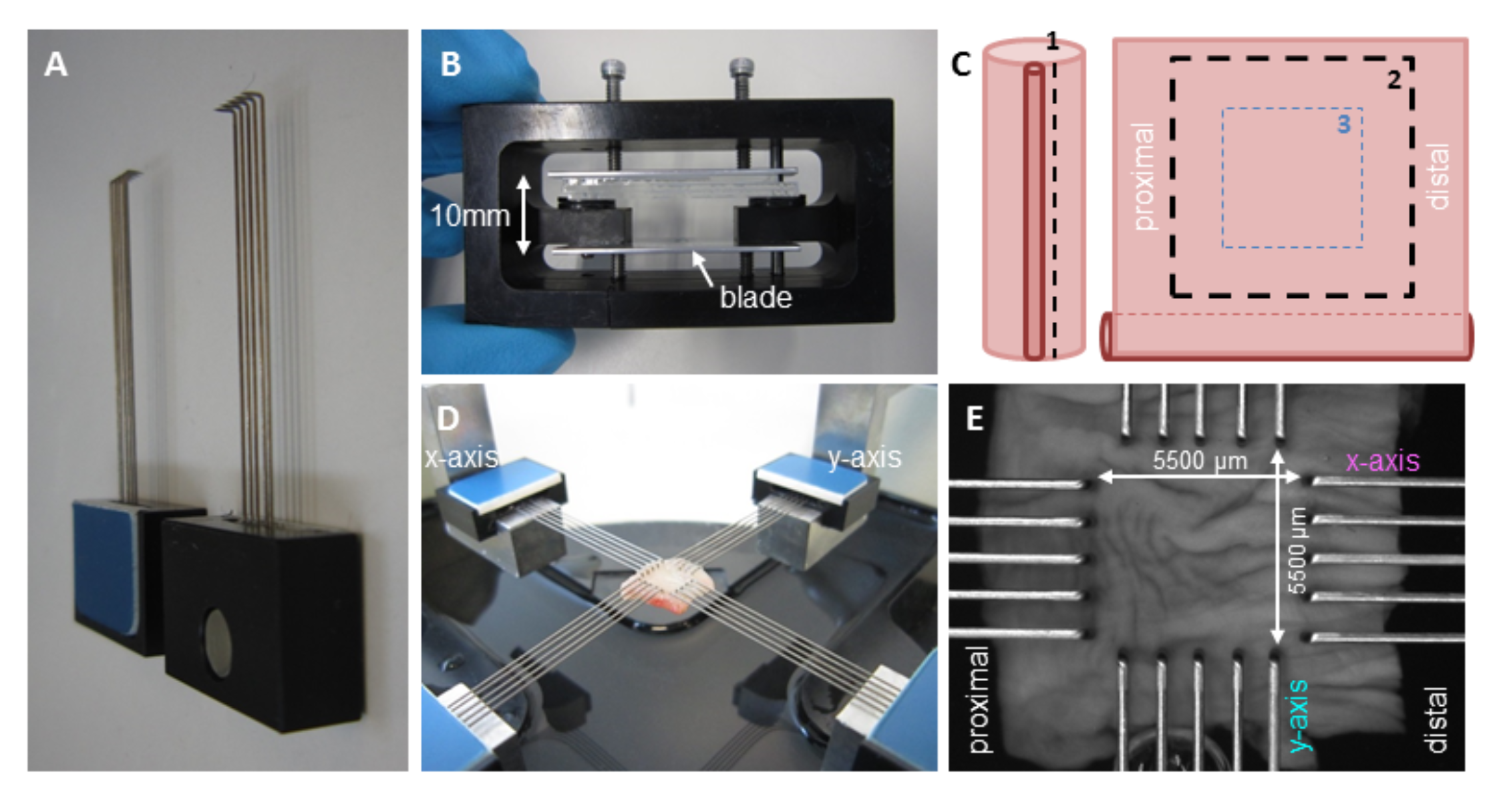

Supplement: Supplementary file 2 — High resolution image (TIF 1900 kb) [file 192_2022_5439_MOESM1_ESM.tif]

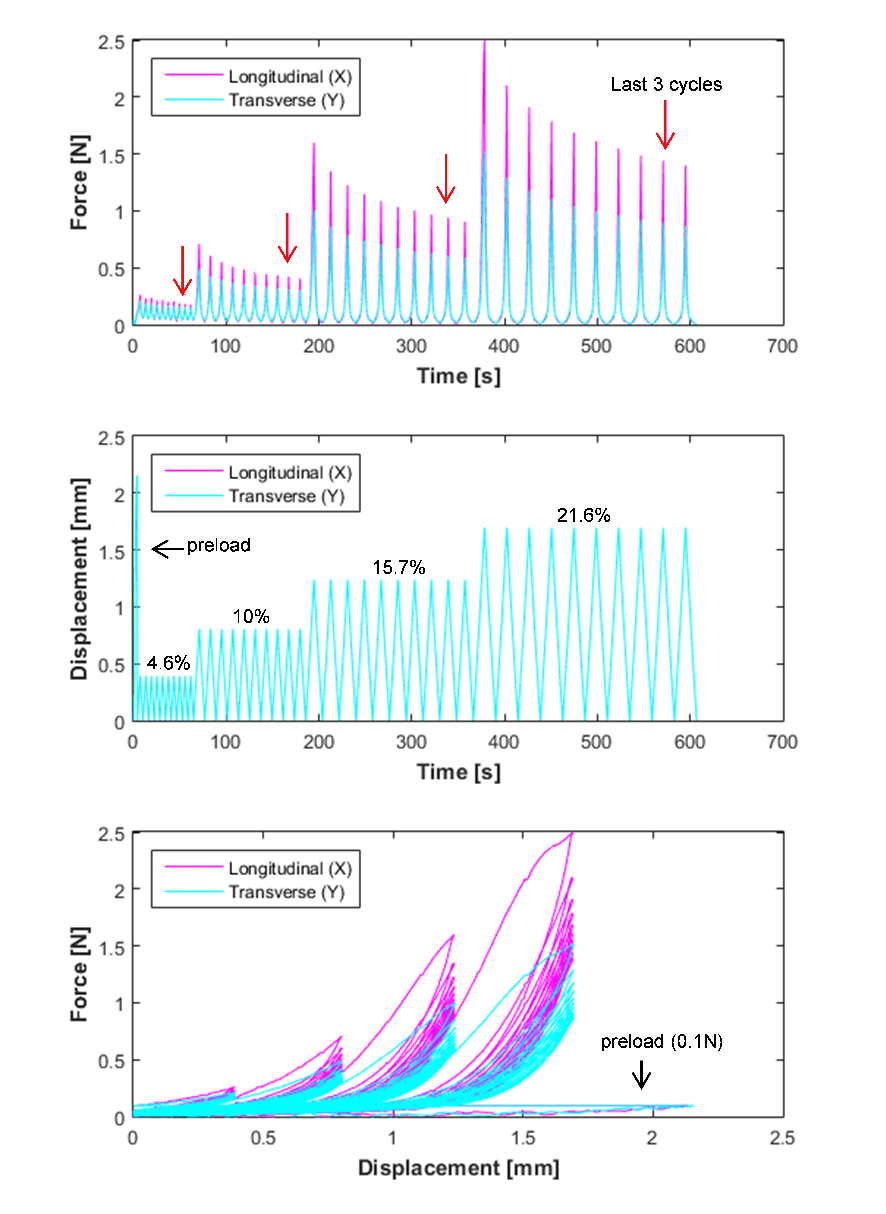

Supplement: Supplementary file 3 — Representative example of a stress–strain curve. All samples demonstrated nonlinear curves in both longitudinal (magenta, x-axis) and transverse (cyan, y-axis) tissue axes. Red arrows indicate the starting point of the last three loading cycles, which exhibit a steady state and were used for further analysis [file 192_2022_5439_Fig7_ESM.png]
